# Development and external validation of an admission-based model for in-hospital mortality in acute exacerbation of COPD: incremental prognostic value of type 2 diabetes mellitus

**DOI:** 10.3389/fendo.2026.1726502

**Published:** 2026-02-11

**Authors:** Jie Chen, Xiaofeng Zhang, Kunhe Liu, Wei Zhang, Mingmei Zhong

**Affiliations:** 1 Department of Respiratory and Critical Care Medicine, The Third Affiliated Hospital of Anhui Medical University (The First People’s Hospital of Hefei), Hefei, China; 2 General Medicine Department, The Second Affiliated Hospital of Anhui Medical University, Hefei, China; 3 Department of Respiratory and Critical Care Medicine, Binhu Hospital of Hefei, Hefei, China; 4 Department of Endocrinology and Metabolism, The Third Affiliated Hospital of Anhui Medical University (The First People’s Hospital of Hefei), Hefei, China

**Keywords:** acute exacerbation, COPD, in-hospital mortality, nomogram, risk prediction, type 2 diabetes mellitus

## Abstract

**Background:**

Acute exacerbation of chronic obstructive pulmonary disease (AECOPD) carries substantial short-term mortality. Whether type 2 diabetes mellitus (T2DM) provides incremental prognostic information for in-hospital death during AECOPD, beyond acute physiological decompensation, remains incompletely defined.

**Methods:**

We conducted a multicenter retrospective cohort study of consecutive inpatients admitted for AECOPD at three tertiary hospitals (June 2022–December 2024). A multivariable logistic model was developed in the training cohort using routinely available variables obtained early after admission, with feature selection by LASSO and performance evaluation by discrimination, calibration, and decision curve analysis. External validation was performed in an independent hospital cohort with intercept/slope recalibration when indicated.

**Results:**

Among 4,292 patients (training n=2,861; external n=1,431), patients with T2DM had higher in-hospital mortality than those without T2DM. In the multivariable model, T2DM contributed incremental prognostic information for in-hospital death (adjusted OR = 2.74, 95% CI 1.62–4.56), together with PaCO_2_, blood urea nitrogen, neutrophil-to-lymphocyte-to-albumin ratio, C-reactive protein, and age. The resulting six-variable nomogram showed strong discrimination (AUC = 0.843 training; 0.817 external), low overall prediction error (external Brier≈0.025), and clinically meaningful net benefit across 5–15% threshold probabilities; calibration in the external cohort was improved to near-ideal after recalibration.

**Conclusion:**

In hospitalized AECOPD, T2DM provides clinically relevant incremental prognostic information for short-term in-hospital mortality within a parsimonious multivariable model. The nomogram may facilitate early risk stratification and support integrated respiratory and metabolic co-management.

## 1 Introduction

Chronic obstructive pulmonary disease (COPD) is a prevalent chronic respiratory disorder and a leading cause of death and disability worldwide (1, 2). Acute exacerbation of COPD (AECOPD) can precipitate rapid deterioration and substantially increase hospitalization, readmission, and short-term mortality risks (3). Accordingly, early admission-based risk stratification is crucial for tailoring monitoring intensity, respiratory support, and resource allocation, yet practical tools for hospitalized AECOPD remain limited—particularly models that integrate metabolic comorbidities with acute physiologic decompensation and demonstrate external transportability.

Type 2 diabetes mellitus (T2DM) is among the most common and clinically actionable comorbidities in COPD; multicenter data suggest that approximately 25%–30% of COPD patients have coexisting T2DM (4). Diabetes has been associated with higher risks of exacerbations and hospitalization (5, 6) and with increased infection susceptibility, dysregulated inflammatory responses, and adverse prognosis (7, 8). During AECOPD, overlapping stressors (infection, hypoxemia or ventilatory impairment, and systemic inflammation) may further amplify T2DM-related immunometabolic dysfunction—via impaired neutrophil function, altered neutrophil extracellular trap (NET) dynamics, and aggravated insulin resistance (7, 9–11)—underscoring the need to quantify its short-term prognostic value and translate it into bedside decision support.

However, key gaps remain: existing evidence is largely association-based and provides limited support for early identification of patients at high risk of in-hospital death; heterogeneity in populations, severity, outcome definitions, and confounding control has produced inconsistent findings and constrained generalizability (12–14); and single-comorbidity associations are insufficient for bedside decisions in this heterogeneous setting. Therefore, we conducted a multicenter retrospective inpatient study using in-hospital mortality as the outcome, developed and externally validated a prediction model based on routinely available early-admission variables, quantified the independent and incremental prognostic contribution of T2DM, and translated the model into a nomogram to support early risk stratification and integrated metabolic-focused management.

## 2 Materials and methods

### 2.1 Study design and data sources

We conducted a multicenter, retrospective, observational cohort study using real-world electronic medical records from three tertiary hospitals (The first people’s Hospital of Hefei, Binhu Hospital of Hefei, and the Second Affiliated Hospital of Anhui Medical University). The cohort included patients admitted with a principal diagnosis of AECOPD between June 2022 and December 2024. According to a prespecified external validation strategy, data from The first people’s Hospital of Hefei and Binhu Hospital of Hefei formed the training cohort (n = 2,861), while data from the Second Affiliated Hospital of Anhui Medical University served as the external validation cohort (n = 1,431). The two datasets did not overlap, yielding a total sample size of 4,292. The primary outcome was in-hospital mortality.

Ethical approval was obtained from the institutional review boards of all participating centers(2025-345-01). Given the retrospective nature of the analysis, written informed consent was waived. All data were anonymized and underwent strict quality control to ensure reliability and clinical generalizability.

### 2.2 Eligibility criteria

Eligible patients were adults aged ≥40 years, regardless of sex, with COPD diagnosed based on medical history and clinical symptoms together with a post-bronchodilator forced expiratory volume in 1 second/forced vital capacity (FEV1/FVC) <70%, or as determined by a respiratory specialist. Patients were required to have a principal admission diagnosis of acute exacerbation of COPD (AECOPD; International Classification of Diseases [ICD] codes J44.100 or J44.000) (15) and key clinical data available within 24 hours of admission (demographics, medical history, and laboratory tests). For individuals with multiple hospitalizations during the study period, only the first admission was included. Patients were excluded if they had comorbid conditions likely to substantially affect respiratory function or short-term prognosis (e.g., active pulmonary tuberculosis, lung cancer, pulmonary fibrosis, bronchiectasis, massive pulmonary embolism, or acute heart failure), or an immunodeficiency state (e.g., HIV/AIDS or long-term use of immunosuppressants).

T2DM was defined by either (i) a prior physician diagnosis and/or long-term use of glucose-lowering medications; or (ii) laboratory confirmation according to the 2024 American Diabetes Association Standards of Care (16) using venous plasma: glycated hemoglobin A1c (HbA1c) ≥6.5%; fasting plasma glucose (FPG; after ≥8 h of fasting) ≥7.0 mmol/L; 2-h plasma glucose ≥11.1 mmol/L during a 75-g oral glucose tolerance test (OGTT); or random plasma glucose ≥11.1 mmol/L in the presence of classic hyperglycemic symptoms or a hyperglycemic crisis. In the absence of classic symptoms or crisis, any positive result required repeat testing on a different day or confirmation by an alternative method.

To distinguish chronic diabetes from transient in-hospital stress- or corticosteroid-related hyperglycemia, patients with isolated hyperglycemia during hospitalization (any glucose value >7.8 mmol/L), no prior evidence of diabetes, and an admission HbA1c <6.5% were not classified as having T2DM. When diabetes status was uncertain (e.g., hyperglycemia during hospitalization with incomplete prior documentation), an endocrinologist reviewed available evidence (prior diagnosis/medication history, admission HbA1c, repeat glucose measurements, and relevant clinical notes) to adjudicate T2DM status; discrepancies were resolved by team discussion. To avoid circularity (incorporation bias) arising from using glycemic indices both to define diabetes status and to predict outcomes, HbA1c and FPG were used only for T2DM ascertainment and glycemic stratification, and were not included as predictors in the primary prognostic model.

### 2.3 Data collection and variables

We collected demographics (age, sex, body mass index [BMI]), disease duration, comorbidities (T2DM, hypertension, coronary heart disease, stroke, heart failure), clinical features (temperature, heart rate, respiratory rate, mean arterial pressure), and laboratory indices (white blood cell count, eosinophil count and percentage, basophil count, hemoglobin, platelet count, alanine aminotransferase, aspartate aminotransferase, neutrophil-to-lymphocyte-to-albumin ratio [NLAR], creatinine, uric acid, blood urea nitrogen [BUN], potassium, sodium, chloride, procalcitonin, C-reactive protein [CRP], N-terminal pro–B-type natriuretic peptide [NT-proBNP], creatine kinase-MB [CK-MB], fasting plasma glucose, HbA1c, D-dimer, pH, arterial partial pressure of oxygen [PaO_2_], arterial partial pressure of carbon dioxide [PaCO_2_], and lactate). Corticosteroid therapy was largely protocolized across centers according to Global Initiative for Chronic Obstructive Lung Disease (3), and dose/duration data were not consistently extractable; therefore, corticosteroid exposure was not included as a model covariate. Stable COPD severity measures (spirometry, COPD Assessment Test, modified Medical Research Council dyspnea scale) were incompletely documented; to mitigate confounding by severity, we adjusted for multiple acute decompensation proxies available within 24 hours of admission. Variable names and units were harmonized across centers. Continuous variables were transformed or standardized after normality testing; categorical variables were factorized before analysis.

### 2.4 Statistical analysis

Continuous variables are presented as mean ± SD or median (IQR), and categorical variables as n (%). Group differences were assessed using the Welch t test or Mann–Whitney U test for continuous variables and χ²/Fisher’s exact tests for categorical variables, as appropriate.

Model development was performed only in the training cohort. Candidate predictors measured within 24 h of admission were screened using LASSO logistic regression with 10-fold cross-validation (λ selected by the 1-SE rule), and selected variables were entered into a multivariable logistic regression model to estimate adjusted ORs with 95% CIs. Discrimination was evaluated by receiver operating characteristic curve (ROC), and calibration by calibration-in-the-large, calibration slope, and calibration curves. Internal validation used bootstrap resampling (B = 1,000) and 10-fold cross-validation to obtain out-of-fold predicted probabilities for decision curve analysis. These resampling procedures were applied to evaluate the final multivariable model (variable selection was not repeated within each resample), and independent external validation was therefore emphasized.

External validation was conducted in an independent cohort by applying the final model without refitting, evaluating area under the ROC curve (AUC), calibration metrics, Brier score, and decision curve analysis; if miscalibration was present, logistic recalibration (intercept and slope) was performed to obtain recalibrated probabilities. Prespecified sensitivity and stratified analyses were performed. Analyses were conducted in R (version 4.4.1), with two-sided P < 0.05.

## 3 Results

### 3.1 Baseline characteristics

**Figure 1:**
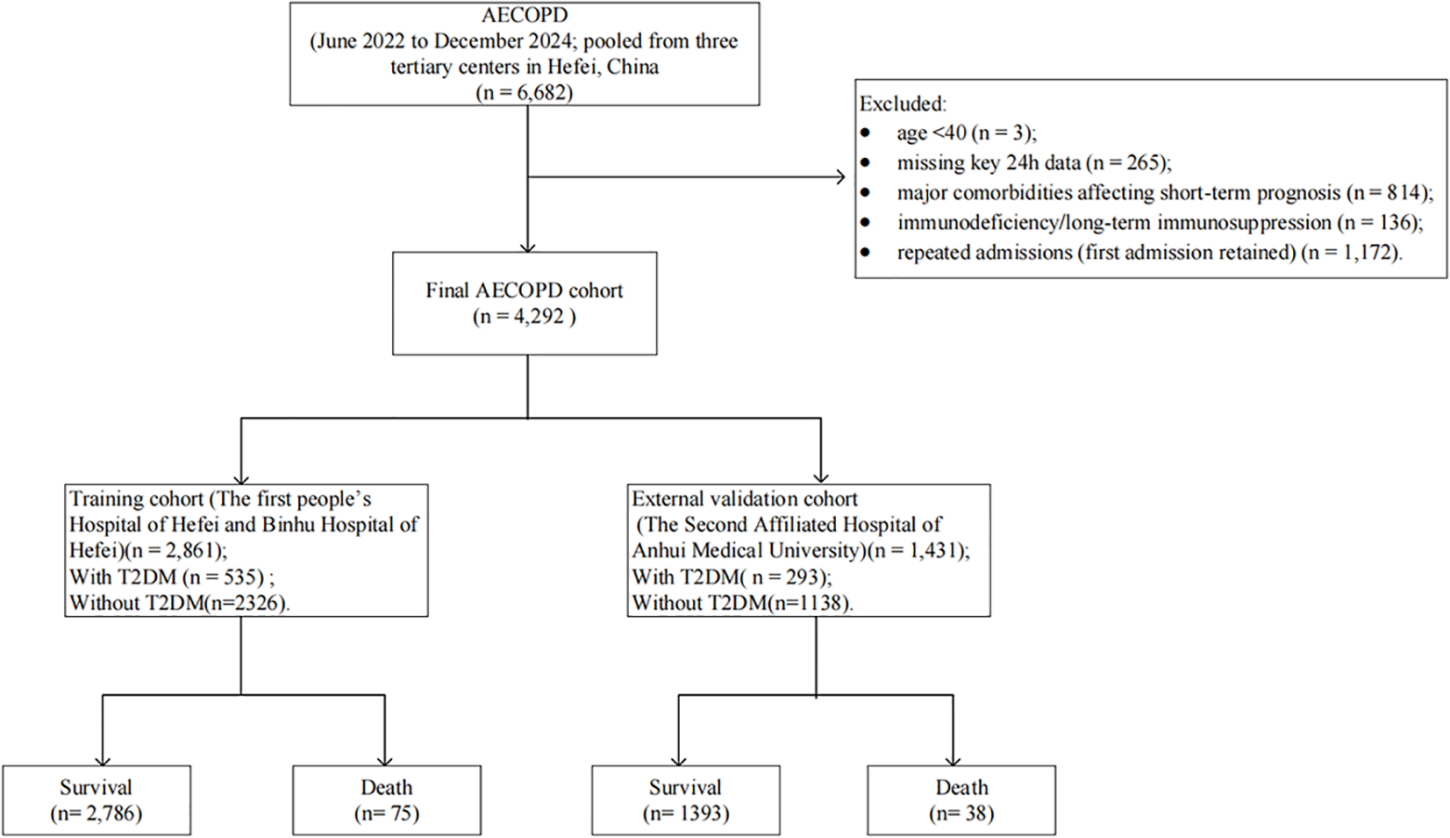
Flowchart.

We finally included 4,292 patients hospitalized with AECOPD: 2,861 in the training cohort and 1,431 in the validation cohort (**Figure 1**). Demographic and baseline characteristics did not differ significantly between the two cohorts (**Supplementary Table S1**).

Patients were grouped by the presence of T2DM. Compared with those without T2DM, patients with T2DM were older (84.00 [74.00–92.00] vs 81.00 [73.00–88.00] years; P<0.001), had a higher BMI (22.58 [20.22–24.76] vs 21.70 [19.07–24.24] kg/m²; P<0.001), and included a greater proportion of women (29.5% vs 24.8%; P = 0.028). Cardiovascular comorbidities were more prevalent in the T2DM group, including hypertension (73.1% vs 47.5%; P<0.001), coronary heart disease (42.8% vs 25.8%; P<0.001), stroke (42.2% vs 22.7%; P<0.001), and heart failure (52.0% vs 35.5%; P<0.001). Several inflammatory/hematologic indices also differed, with higher WBC (7.05 [5.42–8.98] vs 6.47 [5.11–8.30] ×10^9^/L; P = 0.001) and CRP (23.94 [5.00–68.94] vs 18.48 [4.59–55.11] mg/L; P = 0.008), as well as higher eosinophil count (0.11 [0.02–0.21] vs 0.08 [0.01–0.19] ×10^9^/L; P<0.001) and eosinophil percentage (0.02 [0.00–0.04] vs 0.01 [0.00–0.03]; P = 0.005). Admission vital signs and major electrolytes were broadly comparable (e.g., temperature: P = 0.208; sodium: P = 0.129; potassium: P = 0.280), as were coagulation and cardiac/heart failure biomarkers (e.g., D-dimer: P = 0.163; NT-proBNP: P = 0.642; CK-MB: P = 0.496). NLAR as a continuous variable was similar between groups (0.10 [0.06–0.19] vs 0.11 [0.06–0.21]; P = 0.974) (**Table 1**).

**Table 1:** Baseline characteristics of patients with and without T2DM.

Variable	AECOPD without T2DM (n = 2,326)	AECOPD with T2DM (n = 535)	Statistic	P value
Demographics				
Age (years)	81.00 (73.00, 88.00)	84.00 (74.00, 92.00)	532536.5	<0.001
BMI	21.70 (19.07, 24.24)	22.58 (20.22, 24.76)	544078	<0.001
Sex (male)	1,749 (75.2%)	377 (70.5%)	4.845	0.028
Comorbidities				
Hypertension	1,104 (47.5%)	391 (73.1%)	113.413	<0.001
Coronary heart disease	600 (25.8%)	229 (42.8%)	60.317	<0.001
Stroke	527 (22.7%)	226 (42.2%)	85.035	<0.001
Heart failure	826 (35.5%)	278 (52.0%)	48.982	<0.001
Disease duration (years)	2.00 (2.00, 10.00)	7.00 (2.00, 20.00)	518496.5	<0.001
Vital signs				
Temperature (°C)	36.50 (36.30, 36.70)	36.50 (36.30, 36.70)	643751	0.208
Heart rate (beats/min)	84.00 (75.00, 95.00)	82.00 (74.00, 97.00)	642930.5	0.229
Respiratory rate (breaths/min)	20.00 (19.00, 21.00)	20.00 (19.00, 21.00)	618589.5	0.829
Mean arterial pressure (mmHg)	95.33 (86.42, 104.67)	93.67 (84.67, 104.83)	650722	0.098
Laboratory indices				
Fasting plasma glucose (mmol/L)	5.28 (4.52, 6.60)	7.14 (5.42, 9.71)	363737	<0.001
HbA1c (≥ 6.5%)	0 (0.0%)	113 (21.1%)	N/A	N/A
7.35 ≤ pH ≤ 7.45	2,291 (98.5%)	532 (99.4%)	3.141	0.208
pH< 7.35	31 (1.3%)	3 (0.6%)	N/A	N/A
pH> 7.45	4 (0.2%)	0 (0.0%)	N/A	N/A
PaCO_2_ (> 50mmHg)	311 (13.4%)	64 (12.0%)	0.639	0.424
PaO_2_ (< 60mmHg)	104 (4.5%)	37 (6.9%)	5.039	0.025
Lactate (≥ 2mmol/L)	80 (3.4%)	27 (5.0%)	2.691	0.101
WBC (×10^9/L)	6.47 (5.11, 8.30)	7.05 (5.42, 8.98)	562961	0.001
Neutrophils (×10^9/L)	4.39 (3.15, 6.36)	4.72 (3.42, 6.53)	574590.5	0.006
Lymphocytes (×10^9/L)	1.11 (0.76, 1.55)	1.17 (0.81, 1.63)	583307	0.024
Eosinophils (×10^9/L)	0.08 (0.01, 0.19)	0.11 (0.02, 0.21)	552664.5	<0.001
Eosinophil percentage	0.01 (0.00, 0.03)	0.02 (0.00, 0.04)	575355	0.005
Basophils (×10^9/L)	0.02 (0.01, 0.03)	0.02 (0.01, 0.03)	568912.5	0.002
Hemoglobin (g/L)	123.00 (108.00, 136.00)	120.00 (106.00, 133.00)	672696	0.003
C-reactive protein (mg/L)	18.48 (4.59, 55.11)	23.94 (5.00, 68.94)	576251.5	0.008
NLAR	0.11 (0.06, 0.21)	0.10 (0.06, 0.19)	622758	0.974
Platelets (×10^9/L)	180.00 (139.00, 230.00)	192.00 (150.00, 237.00)	569535	0.002
ALT (U/L)	15.00 (10.60, 23.27)	15.80 (10.25, 23.35)	624200	0.908
AST (U/L)	22.00 (16.60, 31.30)	20.10 (15.60, 28.85)	668728.5	0.007
Creatinine (µmol/L)	73.00 (58.00, 91.00)	72.00 (56.00, 93.00)	623821	0.925
BUN (mmol/L)	6.41 (5.01, 8.49)	6.35 (5.00, 8.28)	626327.5	0.811
Uric acid (µmol/L)	277.00 (207.93, 355.00)	267.70 (198.15, 360.50)	635013	0.457
Potassium (mmol/L)	3.98 (3.65, 4.30)	3.98 (3.60, 4.26)	640799	0.28
Sodium (mmol/L)	140.02 (137.49, 142.18)	139.96 (136.87, 142.09)	648367.5	0.129
Chloride (mmol/L)	102.31 (99.74, 105.39)	102.19 (99.30, 104.80)	661762	0.022
Procalcitonin (ng/mL)	0.12 (0.01, 2.90)	0.14 (0.01, 2.64)	619742	0.885
NT-proBNP (pg/mL)	625.03 (100.41, 2,989.77)	594.19 (118.62, 2,448.62)	630189	0.642
CK-MB (U/L)	14.26 (7.29, 22.00)	14.40 (7.50, 23.12)	610486.5	0.496
D-dimer (mg/L)	0.71 (0.35, 1.87)	0.83 (0.38, 1.92)	598192	0.163

Continuous variables are reported as mean ± SD if normally distributed, or median [IQR] otherwise. Categorical variables are n (%). Abbreviations: BMI, body mass index; NLAR, neutrophil-to-lymphocyte-to-albumin ratio; NT-proBNP, N-terminal pro-B-type natriuretic peptide; PaCO_2_, arterial partial pressure of carbon dioxide; PaO_2_, arterial partial pressure of oxygen;WBC, white blood cell count; ALT, alanine aminotransferase; AST, aspartate aminotransferase; CK-MB, creatine kinase-MB. N/A, not applicable or not tested. HbA1c was not tested because it was used in the operational definition of T2DM, to avoid circularity.

### 3.2 In-hospital outcomes

#### 3.2.1 In-hospital mortality

**Figure 2:**
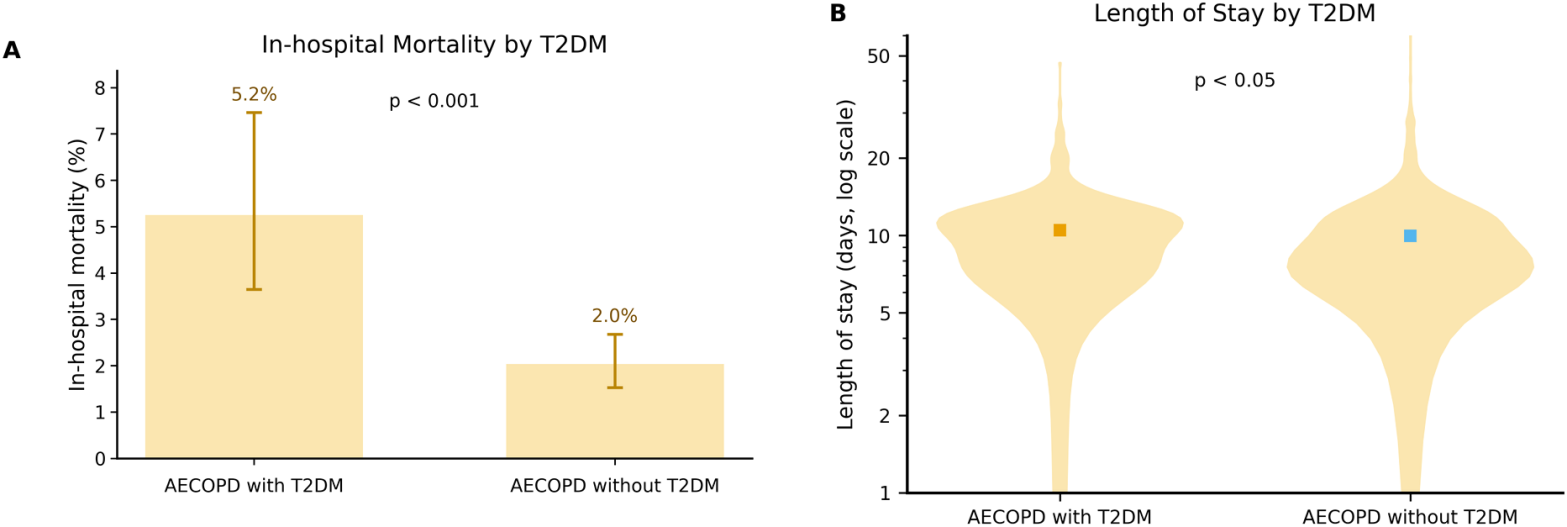
Comparison of in-hospital outcomes between patients with AECOPD with T2DM (n = 535) and AECOPD without T2DM (n = 2326). **(A)** In-hospital mortality in AECOPD with T2DM vs AECOPD without T2DM. Bars indicate observed mortality (%); error bars denote binomial 95% CI (Wilson). The difference between groups was significant (χ²=16.354, P < 0.001; OR = 2.68, 95% CI 1.66–4.32). **(B)** Length of stay in AECOPD with T2DM vs AECOPD without T2DM, assessed with the Welch t-test: t(897.89)=2.255, P < 0.05; mean difference 0.51 days (95% CI 0.07–0.95).

In-hospital mortality differed significantly between groups. Specifically, AECOPD without T2DM patients had 47/2,326 deaths (2.0%), whereas AECOPD with T2DM patients had 28/535 deaths (5.2%). The difference was significant by chi-squared test (χ² = 16.354, P < 0.001), indicating a strong association between concomitant T2DM and increased risk of in-hospital death among AECOPD patients (**Figure 2A**). *Effect size:* OR = 2.68, 95% CI 1.66–4.32.

#### 3.2.2 Length of stay

Length of stay (LOS) also differed significantly. Mean LOS was 9.99 ± 5.28 days in AECOPD without T2DM and 10.50 ± 4.54 days in AECOPD with T2DM patients; the mean difference was 0.51 days (95% CI 0.07–0.95). The Welch t-test was significant (t = 2.255, df = 897.89, P < 0.05) For visualization purposes, LOS is presented on a logarithmic (log10) y-axis in **Figure 2B** to better display the right-skewed distribution and group differences (**Figure 2B**).

### 3.3 Multivariable logistic regression and nomogram development

**Figure 3:**
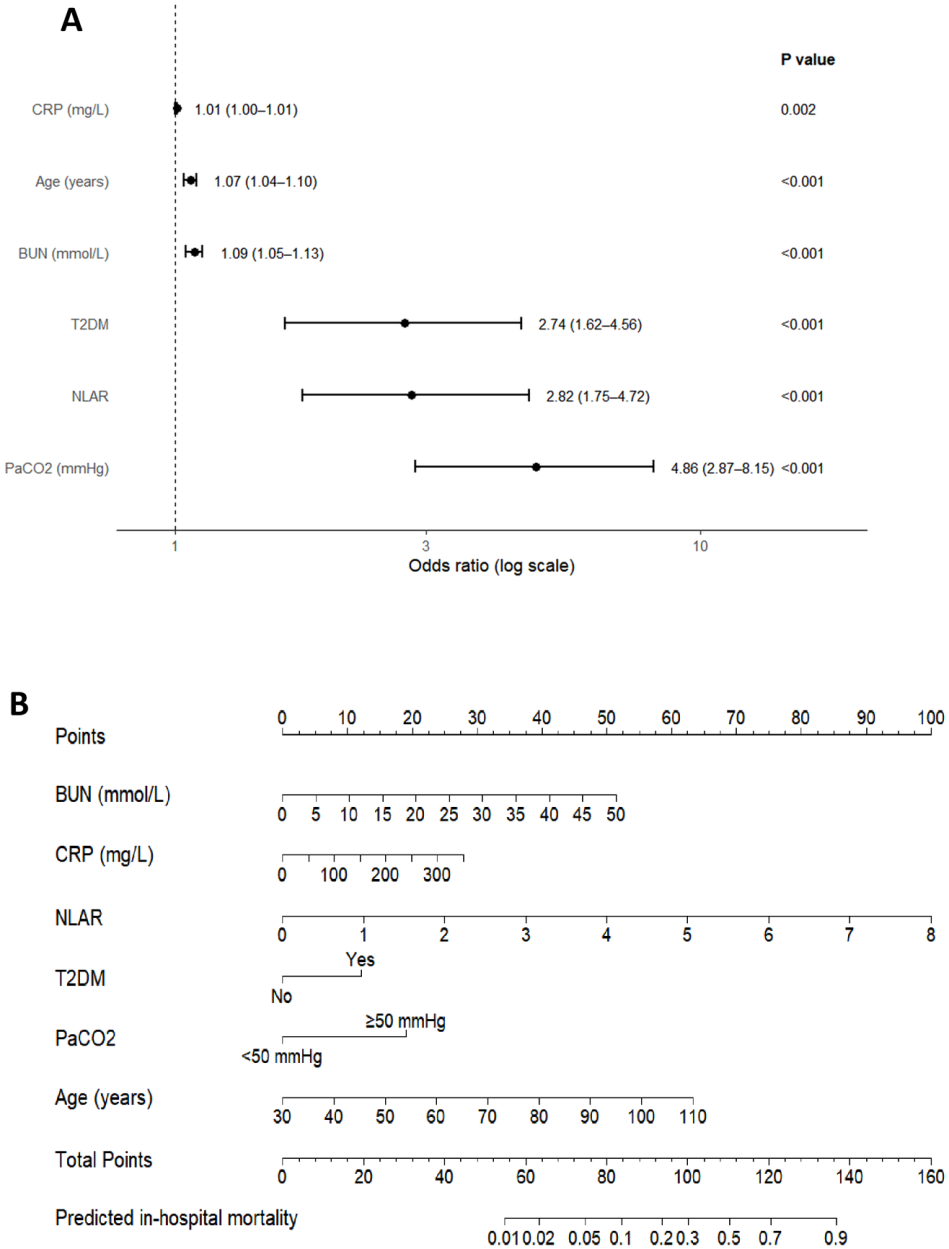
**(A)** Forest plot of the multivariate logistic regression analysis. **(B)** Nomogram of the in-hospital mortality risk model for patients hospitalized with AECOPD. Abbreviations: CRP, C-reactive protein; BUN, blood urea nitrogen; T2DM, type 2 diabetes mellitus; NLAR, Neutrophil-to-Lymphocyte-to-Albumin Ratio; PaCO_2_, arterial partial pressure of carbon dioxide.

In LASSO regression, as the penalty parameter λ increased, some coefficients shrank toward zero (**Supplementary Figure S1A**). Ten-fold cross-validation indicated that models at λ.min and λ.1se both performed well (**Supplementary Figure S1B**). In multivariable logistic regression, PaCO_2_, T2DM, BUN, NLAR, CRP, and age were identified as independent predictors of in-hospital mortality (**Figure 3A**; **Supplementary Table S2**). T2DM remained an independent predictor of in-hospital mortality in AECOPD (OR = 2.74; bootstrap 95% CI 1.55–4.84; Wald 95% CI 1.62–4.56; P < 0.001). Based on the multivariable model, we constructed a nomogram to estimate individual in-hospital mortality risk at admission (**Figure 3B**).To facilitate bedside implementation, we provide a worked example of how to use the nomogram. An 84-year-old patient admitted for AECOPD had BUN 7.6 mmol/L, CRP 35 mg/L, NLAR 0.12 within the first 24 hours, with concomitant T2DM and PaCO_2_ ≥50 mmHg. Entering these values into the nomogram yields an estimated in-hospital mortality risk of approximately 11.84%. This predicted risk falls within a commonly used clinical threshold range (5%–15%), and may support early decisions on intensified monitoring, timely assessment for ventilatory support, and multidisciplinary co-management.

### 3.4 Model performance and internal validation

The model showed excellent discrimination in the training cohort, with an AUC of 0.843. After optimism correction using bootstrap resampling (B = 1,000), the calibration curve closely followed the 45° line, with a Brier score of 0.023 (scaled Brier = 0.093), calibration-in-the-large (CITL) of 0.000, and a calibration slope of 1.000, indicating good overall probability accuracy without apparent overfitting. In K-fold cross-validated decision curve analysis, net benefit consistently exceeded the **“**treat-all**”** and **“**treat-none**”** strategies across clinically relevant thresholds. At threshold probabilities of 5%, 10%, and 15%, the incremental net benefit (ΔNB) was 0.036, 0.088, and 0.149, corresponding to reductions of 67.7, 79.4, and 85.0 unnecessary interventions per 100 patients, respectively (**Supplementary Table S3**).

### 3.5 External validation

**Figure 4:**
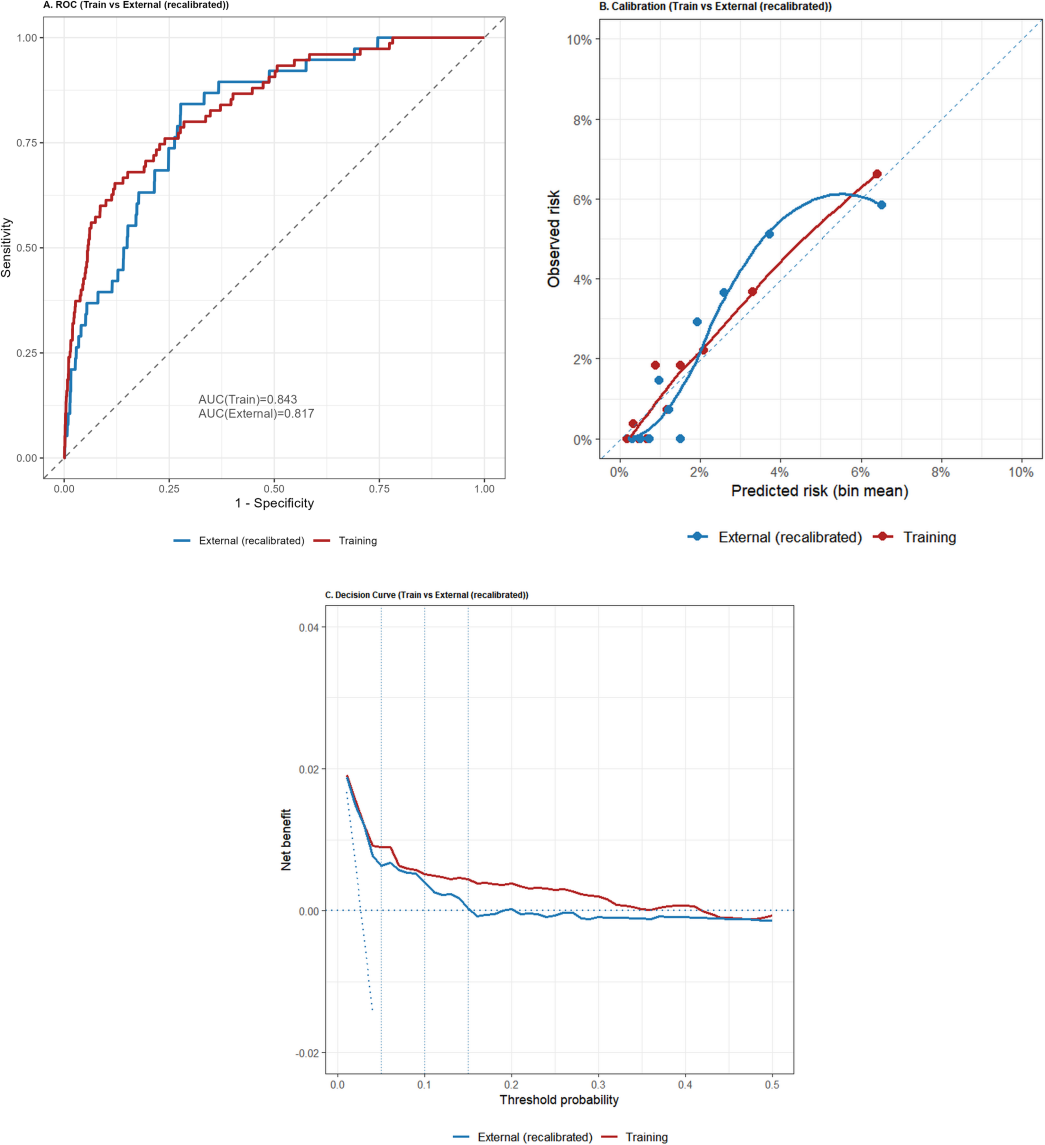
Comparative performance of the training cohort and the external (recalibrated) cohort. **(A)** ROC: The gray dashed line denotes the diagonal reference; AUC(Train) = 0.843 and AUC(External) = 0.817. **(B)** Calibration (0–10%): Decile-binned points with LOESS-smoothed calibration curves; the blue dashed line represents the ideal line (predicted = observed). The external curve is plotted using recalibrated predicted probabilities. **(C)** Decision curve analysis (DCA): Net benefit across threshold probabilities; solid lines are model curves, the horizontal dashed line indicates the treat-none strategy (NB = 0), and vertical dashed lines mark thresholds at 5%, 10%, and 15%. Overall, the recalibrated external cohort shows a net-benefit pattern similar to that of the training cohort, with consistency across commonly used clinical thresholds.

In the external cohort, the AUC was 0.817 (95% CI 0.756–0.878), compared with 0.843 in the training cohort (**Figure 4A**), supporting stable discrimination. Overall prediction error remained low (Brier = 0.0250). Calibration assessment suggested mild miscalibration, with CITL = 0.063 and a calibration slope of 0.883, consistent with a modest systematic offset and slight overfitting when transported to the external population. After logistic recalibration (updating intercept and slope), discrimination was unchanged (AUC = 0.817), with a small improvement in prediction error (Brier = 0.0248) and near-ideal calibration (CITL ≈ 0.000; slope ≈ 1.000) (**Figure 4B**). Decision curve analysis based on recalibrated probabilities demonstrated clinically meaningful utility within the 5%–15% threshold range, yielding an average net benefit (relative to treat-all) of 0.0866—equivalent to 74.30 fewer unnecessary interventions per 100 patients. At thresholds of 5%, 10%, and 15%, ΔNB values were 0.029, 0.085, and 0.147, corresponding to 55.9, 76.8, and 83.1 fewer unnecessary interventions per 100 patients, respectively (**Figure 4C**; **Supplementary Table S4**).

### 3.6 Sensitivity analyses and stratified analyses

To assess the robustness and potential context-dependence of the association between type 2 diabetes mellitus (T2DM) and in-hospital mortality, we performed prespecified sensitivity and stratified analyses (**Supplementary Table S5**). The association remained significant in non-obese patients (BMI < 28 kg/m²; adjusted OR 2.41, 95% CI 1.41–4.12). Stratified analyses suggested heterogeneity by acute severity and inflammatory/infectious burden, with a stronger association among patients with PaCO_2_ ≥ 50 mmHg, whereas the association attenuated in strata with higher inflammatory/infectious load (higher CRP or NLAR quartiles and higher WBC quartiles). Conversely, the association was more pronounced in the lower WBC strata (Q1–Q2; adjusted OR 7.07 and 4.43, respectively). In addition, within the T2DM subgroup (n=535), HbA1c ≥ 6.5% (n=113) versus < 6.5% (n=422) was not significantly associated with in-hospital mortality (3/113 vs 25/422; Fisher’s exact P = 0.234; OR 0.43, 95% CI 0.13–1.46), suggesting that the observed prognostic signal of T2DM is unlikely to be fully attributable to baseline HbA1c strata, although this exploratory analysis was limited by sparse events.

## 4 Discussion

In this multicenter inpatient AECOPD cohort, we assessed the prognostic association between T2DM and in-hospital mortality and developed a risk model composed of routinely available variables within the first 24 hours of admission. Patients with T2DM had higher mortality and slightly longer length of stay. In multivariable analyses, T2DM retained independent prognostic value beyond PaCO_2_, BUN, CRP, NLAR, and age, suggesting that it may contribute to early risk stratification at admission. The model demonstrated good discrimination in both the training and external validation cohorts, with acceptable calibration and clinical net benefit. Beyond discrimination, calibration metrics indicate whether predicted probabilities are clinically reliable. In external validation, the low Brier score (0.0250) suggests small overall prediction error, whereas CITL = 0.063 and a slope of 0.883 indicate mild offset and modest overfitting during transport; importantly, logistic recalibration restored near-ideal calibration (CITL ≈ 0; slope ≈ 1.0) without loss of discrimination.

PaCO_2_ contributed a larger effect estimate in the model, which is clinically plausible because short-term inpatient death during AECOPD is often driven by ventilatory failure and acute decompensation (17). At the same time, the prominence of acute markers should not be interpreted as making comorbidities clinically irrelevant. Acute physiology primarily reflects the intensity of the current exacerbation, whereas T2DM may serve as a readily identifiable marker of broader comorbidity burden and reduced physiological reserve. We focused on T2DM because it is common in COPD (18), is often underestimated in exacerbation-risk evaluation (19), and is readily identifiable with well-established intervention pathways; therefore, incorporating T2DM into an early risk model may help refine risk stratification and support coordinated respiratory–metabolic care during hospitalization.

T2DM may exacerbate disease severity in AECOPD and increase mortality risk through multiple pathways: (1) impaired immune defense, whereby hyperglycemia compromises host responses to pathogens and increases the risk of respiratory infections (20); (2) amplified systemic inflammation, as patients with T2DM tend to have higher baseline inflammatory activity and may mount more intense inflammatory responses during AECOPD, accelerating lung injury and multi-organ dysfunction (20); (3) microvascular dysfunction and tissue hypoxia, whereby chronic dysglycemia induces endothelial dysfunction and impaired capillary perfusion, reducing tolerance to hypoxemia and hypercapnia (21); (4) a metabolic–inflammatory vicious cycle, in which chronic inflammation and oxidative stress in AECOPD aggravate insulin resistance, while hyperglycemia further promotes inflammatory mediator release (20, 22); and (5) treatment-related hyperglycemia, since systemic corticosteroids commonly used in AECOPD can induce or worsen hyperglycemia and increase metabolic stress in patients with T2DM (23). The convergence of these mechanisms may help explain the significant association between T2DM and mortality observed in our study.

Sensitivity and subgroup analyses further contextualized the prognostic implications of T2DM. First, the association between T2DM and in-hospital mortality remained significant in non-obese patients (BMI < 28 kg/m²), suggesting that the signal was not entirely driven by obesity-related phenotypes, consistent with Li et al. (24). Second, stratified by severity, the association was stronger in patients with PaCO_2_ ≥ 50 mmHg, whereas it tended to attenuate or become non-significant in strata with higher inflammatory burden (higher CRP/NLAR), implying that the prognostic signal of T2DM may vary with acute physiological and inflammatory context (25, 26). In addition, within the T2DM subgroup, no difference in in-hospital mortality was observed when stratified by HbA1c (≥6.5% vs. <6.5%) (exploratory analysis with limited events), suggesting that the short-term risk information captured by T2DM in our cohort may not be fully explained by chronic glycemic control as reflected by HbA1c alone (27). Collectively, these findings reinforce a pragmatic clinical message: T2DM is neither a “substitute” for acute severity indicators nor a negligible background label; rather, it may provide additional risk signals under specific clinical contexts.

By focusing on AECOPD with comorbid T2DM—a common, high-risk, and highly heterogeneous subgroup—we developed a targeted model composed of routinely available early-admission variables. The model is rapid, interpretable, and low in measurement cost, enabling more agile identification of high-risk individuals and more rational allocation of monitoring and treatment resources in the emergency department and early during hospitalization.

This study also has limitations. First, retrospective electronic medical record analyses may introduce misclassification of diabetes; although we applied a prespecified algorithm and had endocrinologists adjudicate challenging cases to distinguish chronic T2DM from stress- or glucocorticoid-related hyperglycemia during hospitalization, residual misclassification cannot be fully excluded. Second, data on corticosteroid dose/duration and stable COPD severity (spirometry, CAT, mMRC) were incompletely captured in inpatient records. Although we adjusted for multiple proxies of acute severity/systemic stress within the first 24 hours after admission, residual confounding related to baseline severity and treatment intensity may remain; therefore, findings should be interpreted within an association-based prognostic framework. Third, the external validation cohort was geographically proximate with similar practice patterns, potentially limiting transportability to other regions and healthcare systems. Finally, subgroup and sensitivity analyses were exploratory; sparse events in some strata and inconsistent coding of ventilatory support (NIV/IMV) reduced precision and limited finer-grained severity stratification.

Future prospective, multi-regional studies are warranted to standardize the collection of key information—including ventilatory support, corticosteroid exposure, stable-phase severity, and post-discharge outcomes—to further evaluate model generalizability and facilitate clinical translation. Moreover, incorporating follow-up data at 90 days, 1 year, and longer horizons may clarify the value of the model for predicting longer-term readmission and post-discharge mortality. With the continued evolution of artificial intelligence, integrating biomarkers, clinical history, and imaging into a more refined multimodal model may further improve predictive accuracy and individualized risk stratification.

## 5 Conclusion

T2DM is an independent prognostic factor for in-hospital mortality among patients admitted with AECOPD. The proposed model demonstrates good discrimination, calibration, and clinical utility in both internal and external validation. Incorporating T2DM into AECOPD risk assessment can facilitate early identification of high-risk patients and guide individualized management strategies to improve outcomes.

## Data Availability

The original contributions presented in the study are included in the article/**Supplementary Material**. Further inquiries can be directed to the corresponding author.
