# Supplementary material for: Development and external validation of an admission-based model for in-hospital mortality in acute exacerbation of COPD: incremental prognostic value of type 2 diabetes mellitus
Source: Front Endocrinol (Lausanne). 2026 Feb 11;17:1726502. doi: 10.3389/fendo.2026.1726502 (PMC12932170; doi:10.3389/fendo.2026.1726502)

**Table S1.** Baseline demographics and characteristics of all patients in the AECOPD training and validation cohorts.

| **Variable** | **Category** | **Training group（n=2861）** | **Validation Group(n=1431)** | **Statistic** | **P value** |
| --- | --- | --- | --- | --- | --- |
| Sex(%) | Female | 735 (25.7%) | 363 (25.4%) | 0.052 | 0.819 |
|  | Male | 2126 (74.3%) | 1068 (74.6%) |  |  |
| BMI |  | 22.0(19.2,24.5) | 21.8(19.1,24.5) | 2064454 | 0.649 |
| Age (years) |  | 81.0(73.0,89.0) | 81.0(74.0,89.0) | 2041460 | 0.884 |
| Disease duration (years) |  | 3.0(2.0,13.0) | 3.0(2.0,15.0) | 2020298 | 0.463 |
| T2DM(%) | No | 2326 (81.3%) | 1138 (79.5%) | 1.931 | 0.165 |
|  | Yes | 535 (18.7%) | 293 (20.5%) |  |  |
| Hypertension(%) | No | 1366 (47.7%) | 717 (50.1%) | 2.126 | 0.145 |
|  | Yes | 1495 (52.3%) | 714 (49.9%) |  |  |
| Coronary heart disease(%) | No | 2032 (71.0%) | 1006 (70.3%) | 0.242 | 0.623 |
|  | Yes | 829 (29.0%) | 425 (29.7%) |  |  |
| Stroke(%) | No | 2108 (73.7%) | 1034 (72.3%) | 0.985 | 0.321 |
|  | Yes | 753 (26.3%) | 397 (27.7%) |  |  |
| Heart failure(%) | No | 1757 (61.4%) | 893 (62.4%) | 0.397 | 0.529 |
|  | Yes | 1104 (38.6%) | 538 (37.6%) |  |  |
| HbA1c （≥6.5） | No | 2748 (96.1%) | 1365 (95.4%) | 1.048 | 0.306 |
| (%) | Yes | 113 (3.9%) | 66 (4.6%) |  |  |
| Fasting plasma glucose (mmol/L) |  | 5.5(4.6,7.1) | 5.5(4.5,7.1) | 2059474 | 0.745 |
| pH(%) | 7.35-7.45 | 2823 (98.7%) | 1415 (98.9%) | 0.569 | 0.752 |
|  | ＜7.35 | 34 (1.2%) | 15 (1.0%) |  |  |
|  | ＞7.45 | 4 (0.1%) | 1 (0.1%) |  |  |
| PaCO_2_（≥50mmHg） | No | 2486 (86.9%) | 1234 (86.2%) | 0.359 | 0.549 |
| (%) | Yes | 375 (13.1%) | 197 (13.8%) |  |  |
| PaO_2_（≥60mmHg） | No | 151 (5.3%) | 96 (6.7%) |  |  |
| (%) | Yes | 2710 (94.7%) | 1335 (93.3%) | 3.6 | 0.058 |
| Lactate(≥2mmol/L） | No | 2754 (96.3%) | 1365 (95.4%) | 1.876 | 0.171 |
| (%) | Yes | 107 (3.7%) | 66 (4.6%) |  |  |
| Temperature (℃) |  | 36.5(36.3,36.7) | 36.5(36.3,36.8) | 1990112 | 0.134 |
| Heart rate (beats/min) |  | 84.0(75.0,96.0) | 83.0(75.0,95.0) | 2069338 | 0.560 |
| Respiratory rate (breaths/min) |  | 20.0(19.0,21.0) | 20.0(19.0,22.0) | 2026224 | 0.576 |
| Mean arterial pressure (mmHg) |  | 95.0(86.0,104.7) | 95.3(86.3,104.2) | 2037497 | 0.803 |
| WBC (×10^9/L) |  | 6.5(5.1,8.4) | 6.8(5.2,8.6) | 1986496 | 0.114 |
| Neutrophils (×10^9/L) |  | 4.5(3.2,6.4) | 4.6(3.3,6.4) | 1990758 | 0.141 |
| Lymphocytes (×10^9/L) |  | 1.1(0.8,1.6) | 1.1(0.8,1.6) | 1985430 | 0.107 |
| Eosinophils (×10^9/L) |  | 0.1(0.0,0.2) | 0.1(0.0,0.2) | 2091769 | 0.241 |
| Basophils (×10^9/L) |  | 0.0(0.0,0.0) | 0.0(0.0,0.0) | 2086772 | 0.288 |
| Hemoglobin (g/L) |  | 122.0(108.0,135.0) | 121.0(107.0,135.0) | 2060924 | 0.717 |
| Platelets (×10^9/L) |  | 181.0(141.0,231.0) | 183.0(142.0,228.0) | 2055289 | 0.830 |
| ALT (U/L) |  | 15.1(10.5,23.3) | 15.2(10.8,23.3) | 2029027 | 0.638 |
| AST (U/L) |  | 21.8(16.3,30.8) | 21.4(16.8,34.0) | 1987690 | 0.121 |
| Creatinine (µmol/L) |  | 73.0(58.0,91.2) | 74.0(58.0,92.0) | 2038608 | 0.826 |
| BUN (mmol/L) |  | 6.4(5.0,8.4) | 6.4(5.0,8.4) | 2062152 | 0.693 |
| Potassium (mmol/L) |  | 4.0(3.6,4.3) | 4.0(3.7,4.3) | 1992286 | 0.153 |
| Sodium (mmol/L) |  | 140.0(137.4,142.2) | 140.0(137.3,142.2) | 2060254 | 0.730 |
| NT-proBNP（pg/mL） |  | 619.3(103.0,2947.8) | 549.8(83.8,2799.1) | 2103001 | 0.143 |
| CK‑MB (U/L) |  | 14.3(7.3,22.2) | 13.8(6.9,21.4) | 2109583 | 0.102 |
| D‑dimer (mg/L) |  | 0.7(0.4,1.9) | 0.7(0.3,1.8) | 2052291 | 0.891 |
| NLAR |  | 0.1(0.1,0.2) | 0.1(0.1,0.2) | 2032836 | 0.710 |
| Albumin(g/L) |  | 37.1±5.0 | 37.0±5.0 | 0.552 | 0.581 |
| C-reactive protein (mg/L) |  | 19.3(4.7,57.5) | 6.1(4.0,51.4) | 2113154 | 0.084 |
| Procalcitonin (ng/mL) |  | 0.1(0.0,2.8) | 0.1(0.0,2.6) | 2069328 | 0.557 |

**Note:** Continuous variables are reported as mean ± standard deviation if normally distributed, or median [IQR] otherwise. Categorical variables are presented as n (%). Abbreviations: NLAR = neutrophil-to-lymphocyte ratio divided by albumin; NT-proBNP = N-terminal pro–B-type natriuretic peptide; pH = acidity/alkalinity; PaCO₂ = arterial partial pressure of carbon dioxide; PaO₂ = arterial partial pressure of oxygen.

**Table S2.** Multivariable logistic regression analysis of in-hospital mortality in AECOPD.

| **term** | **estimate** | **conf.low** | **conf.high** | **p.value** |
| --- | --- | --- | --- | --- |
| (Intercept) | 0.000015 | 0.000000893 | 0.000194 | 0.000 |
| BUN | 1.09 | 1.05 | 1.13 | 0.000 |
| CRP | 1.01 | 1 | 1.01 | 0.002 |
| NLAR | 2.82 | 1.75 | 4.72 | 0.000 |
| T2DM | 2.74 | 1.62 | 4.56 | 0.000 |
| PaCO_2_ | 4.86 | 2.87 | 8.15 | 0.000 |
| Age | 1.07 | 1.04 | 1.1 | 0.000 |

Abbreviations: BUN, blood urea nitrogen; CRP, C-reactive protein; NLAR, neutrophil-to-lymphocyte ratio divided by albumin; T2DM, type 2 diabetes mellitus; PaCO₂, arterial partial pressure of carbon dioxide.

**Table S3.** Decision curve analysis (DCA) under K-fold cross-validation

| **threshold** | **DeltaNB** | **DeltaNB_L** | **DeltaNB_U** | **NR_per100** | **NR_L** | **NR_U** |
| --- | --- | --- | --- | --- | --- | --- |
| 0.0500 | 0.0356 | 0.0314 | 0.0393 | 67.6686 | 59.7344 | 74.6601 |
| 0.1000 | 0.0882 | 0.0831 | 0.0931 | 79.4128 | 74.7981 | 83.7819 |
| 0.1500 | 0.1492 | 0.1434 | 0.1548 | 84.5625 | 81.2531 | 87.7083 |

probability; ΔNB = relative net benefit; NR = net reduction in unnecessary interventions per 100 patients.

**Table S4.** Decision curve analysis (DCA) for external validation (using recalibrated probabilities).

| **threshold** | **DeltaNB** | **DeltaNB_L** | **DeltaNB_U** | **NR_per100** | **NR_L** | **NR_U** |
| --- | --- | --- | --- | --- | --- | --- |
| 0.0500 | 0.0294 | 0.0224 | 0.0364 | 55.9050 | 42.5559 | 69.1841 |
| 0.1000 | 0.0853 | 0.0773 | 0.0929 | 76.7994 | 69.5300 | 83.6478 |
| 0.1500 | 0.1467 | 0.1377 | 0.1556 | 83.1353 | 78.0090 | 88.1901 |

**Note:** Abbreviations: pt = threshold probability; ΔNB = relative net benefit; NR = net reduction in unnecessary interventions per 100 patients.

| **Table S5. Sensitivity and stratified analyses of the association between T2DM and in-hospital mortality in AECOPD.** | | | | | | | | |
| --- | --- | --- | --- | --- | --- | --- | --- | --- |
| **Subgroup/Stratum** | **N** | **Deaths** | **T2DM deaths/n** | **Non-T2DM deaths/n** | **Crude OR (95% CI)** | **P (crude)** | **Adjusted OR (95% CI)** | **P (adjusted)** |
| **A. Sensitivity analyses** |  |  |  |  |  |  |  |  |
| Non-obese (BMI < 28 kg/m²) | 2688 | 70 | 25/495 | 45/2193 | 2.54 (1.54–4.18) | <0.001 | 2.41 (1.41–4.12) | <0.01 |
| No hypertension | 1366 | 32 | 6/144 | 26/1222 | 2.00 (0.81–4.94) | 0.133 | 1.31 (0.46–3.72) | 0.610 |
| T2DM subgroup: HbA1c ≥6.5% vs <6.5% | 535 | 28 | 3/113 | 25/422 | 0.43 (0.13–1.46) | 0.234 |  |  |
|  |  |  |  |  |  |  |  |  |
| **B. Stratified analyses by severity and inflammatory burden** |  |  |  |  |  |  |  |  |
| PaCO₂ category |  |  |  |  |  |  |  |  |
| <50 mmHg | 2486 | 46 | 18/471 | 28/2015 | 2.82 (1.55–5.14) | 0.002 | 2.41 (1.27–4.58) | 0.007 |
| ≥50 mmHg | 375 | 29 | 10/64 | 19/311 | 2.85 (1.25–6.45) | 0.018 | 4.10 (1.64–10.28) | 0.003 |
|  |  |  |  |  |  |  |  |  |
| Arterial pH category |  |  |  |  |  |  |  |  |
| Normal | 2823 | 75 | 28/532 | 47/2291 | 2.65 (1.64–4.28) | <0.001 | 2.73 (1.63–4.57) | <0.001 |
| Low | 38 | 0 | 0/3 | 0/35 | 10.14 (0.17–594.42) | 1.000 |  |  |
|  |  |  |  |  |  |  |  |  |
| ICU admission |  |  |  |  |  |  |  |  |
| Non-ICU | 2652 | 45 | 8/502 | 37/2150 | 0.92 (0.43–2.00) | 1.000 | 0.75 (0.32–1.73) | 0.494 |
| ICU | 209 | 30 | 20/33 | 10/176 | 25.54 (9.92–65.77) | <0.001 | 51.62 (15.76–169.07) | <0.001 |
|  |  |  |  |  |  |  |  |  |
| CRP quartiles |  |  |  |  |  |  |  |  |
| Q1 | 716 | 6 | 5/126 | 1/590 | 24.34 (2.82–210.19) | <0.001 | 27.00 (2.81–259.19) | 0.004 |
| Q2 | 715 | 15 | 5/116 | 10/599 | 2.65 (0.89–7.91) | 0.080 | 2.87 (0.88–9.28) | 0.079 |
| Q3 | 716 | 19 | 9/139 | 10/577 | 3.93 (1.56–9.85) | 0.005 | 2.75 (1.00–7.55) | 0.050 |
| Q4 | 714 | 35 | 9/154 | 26/560 | 1.27 (0.58–2.78) | 0.530 | 1.44 (0.62–3.37) | 0.395 |
|  |  |  |  |  |  |  |  |  |
| NLAR quartiles |  |  |  |  |  |  |  |  |
| Q1 | 716 | 15 | 10/122 | 5/594 | 10.52 (3.53–31.36) | <0.001 | 12.14 (3.55–41.52) | <0.001 |
| Q2 | 715 | 7 | 2/156 | 5/559 | 1.44 (0.28–7.49) | 0.650 | 1.20 (0.21–6.77) | 0.834 |
| Q3 | 715 | 8 | 5/135 | 3/580 | 7.40 (1.75–31.35) | 0.008 | 10.09 (2.22–45.88) | 0.003 |
| Q4 | 715 | 45 | 11/122 | 34/593 | 1.63 (0.80–3.31) | 0.216 | 1.37 (0.64–2.95) | 0.416 |
| WBC quartiles |  |  |  |  |  |  |  |  |
| Q1 | 716 | 14 | 7/110 | 7/606 | 5.82 (2.00–16.93) | 0.001 | 7.07 (2.22–22.48) | <0.001 |
| Q2 | 715 | 22 | 10/125 | 12/590 | 4.19 (1.77–9.93) | 0.001 | 4.43 (1.68–11.66) | 0.003 |
| Q3 | 715 | 15 | 5/147 | 10/568 | 1.96 (0.66–5.84) | 0.224 | 1.86 (0.57–6.04) | 0.305 |
| Q4 | 715 | 24 | 6/153 | 18/562 | 1.23 (0.48–3.16) | 0.662 | 1.14 (0.39–3.31) | 0.812 |
| Abbreviations: AECOPD, acute exacerbation of chronic obstructive pulmonary disease; BMI, body mass index; BUN, blood urea nitrogen; CITL, calibration-in-the-large; CRP, C-reactive protein; ICU, intensive care unit; NLAR, neutrophil-to-lymphocyte-to-albumin ratio; OR, odds ratio; PaCO₂, partial pressure of arterial carbon dioxide; T2DM, type 2 diabetes mellitus; WBC, white blood cell count. | | | | | | | | |
|  |  |  |  |  |  |  |  |  |

Supplementary Fig S1. (A) LASSO coefficient trajectory plot showing the variation of candidate predictor coefficients under different log(λ) values. (B) Ten-fold cross-validation curve of the LASSO model.


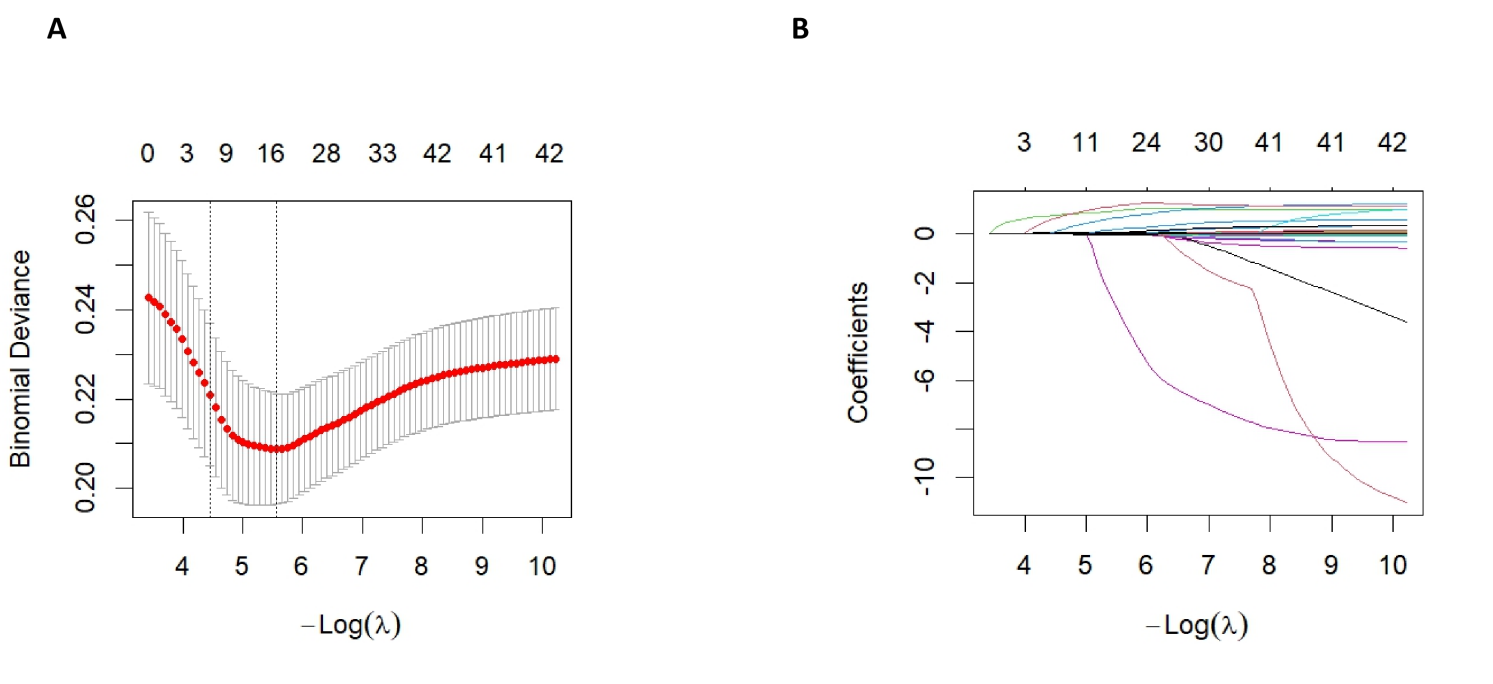

Supplement: Supplementary file 1 [file DataSheet1.docx]
